# Profound deficiencies in mature blood and bone marrow progenitor dendritic cells in Chronic Lymphocyticcytic Leukemia patients

**DOI:** 10.21203/rs.3.rs-4953853/v1

**Published:** 2024-09-27

**Authors:** Baustin M. Welch, Sameer A. Parikh, Neil E. Kay, Kay L. Medina

**Affiliations:** 1 Department of Immunology, Mayo Clinic, Rochester, MN 55905, USA; 2 Mayo Clinic Graduate School of Biomedical Sciences, Mayo Clinic, Rochester, MN 55905, USA; 3 Division of Hematology, Mayo Clinic, Rochester, MN 55905, USA

## Abstract

Chronic lymphocytic leukemia (CLL) patients are immunocompromised and highly vulnerable to serious recurrent infections. Conventional dendritic cells (cDCs) and plasmacytoid DCs (pDCs) are principal sensors of infection and are essential in orchestrating innate and adaptive immune responses to resolve infection. This study identified significant deficiencies in six functionally distinct DC subsets in blood of untreated CLL (UT-CLL) patients and selective normalization of pDCs in response to acalabrutinib (a Bruton tyrosine kinase inhibitor) therapy. DCs are continuously replenished from hematopoiesis in bone marrow (BM). Four BM developmental intermediates that give rise to cDCs and pDCs were examined and significant reductions of these were identified in UT-CLL patients supporting a precursor/progeny relationship. The deficiencies in blood DCs and BM DC progenitors were significantly associated with alterations in the Flt3/FL signaling pathway critical to DC development and function. Regarding clinical parameter, cDC subset deficiencies are associated with adverse prognostic indicators of disease progression, including *IGHV* mutation, CD49d, CD38, and ZAP-70 status. Importantly, UT-CLL patients with shared DC subset deficiencies had shorter time-to-first treatment (TTFT), uncovering a profound alteration in innate immunity with the potential to instruct therapeutic decision-making.

## Introduction

Chronic lymphocytic leukemia (CLL) accounts for approximately one-third of newly diagnosed leukemias (1). In CLL, malignant clonal CD19^+^ CD5^+^ B cells accumulate in blood, bone marrow (BM), and secondary lymphoid tissues (2). Besides the array of leukemic manifestations, many CLL patients are afflicted with higher rates of viral and bacterial infections, the latter being a leading cause of morbidity and mortality (3–5). Increased susceptibility to infection reflects an underlying global immunodeficiency of CLL hallmarked by diminished ability to successfully resolve immunologic challenges (6–11). CLL patients also have impaired BM hematopoiesis, which may be the root cause of some immunodeficiencies (12, 13). A major gap in knowledge of the CLL immune system is the status and function of dendritic cells (DCs). However, there have been major advances in defining both the array of DC subsets and their function.

Initiating competent immune responses to pathogens requires the interconnection of innate and adaptive immunity. DCs fulfill this pivotal role by continuously surveying the microenvironment for immunological perturbations (14). Recently, human DC subsets have been more precisely defined. Factors such as ontogeny, phenotype, function, and tissue localization have clarified what are considered *bona fide* DCs, i.e., conventional DCs (cDCs) (15). cDCs develop from hematopoietic stem and progenitor cells (HSPCs) in BM that differentiate into common DC progenitors (CDPs) and subsequently pre-cDCs, which give rise to cDCs (15–17). In tissues, mature cDCs act as sentinels and upon detection of danger signals they traffic to lymph nodes (LNs) to prime antigen-specific naïve T cells (18). Plasmacytoid DCs (pDCs) are distinct from cDCs and also stem from CDPs (16).

DCs highly express MHC class II. cDC (CD11c^+^ CD123^−^) subpopulations (cDC1 and cDC2) exhibit differential Toll-like receptor (TLR) and C-type lectin receptor (CLR) expression, localization, and functions (19–21). cDC1, expressing CLEC9A and CD141, are superior cross-presenters of extracellular antigens on MHC class I to CD8^+^ T cells, which is critical in anti-tumor immunity (15, 22–26). cDC2s express CD1c, SIRPα, CLEC10A and are comprised of functionally distinct CD5^−^ and CD5^+^ subpopulations (27–29). pDCs are CD11c^−^ CD123^+^ CD303^+^, secrete high levels of type I IFNs, and aid in anti-viral responses (30). Recently, a DC expressing Axl and Siglec-6 (AS DC) was identified (31, 32). Axl is a member of the TAM family (Tyro3, Axl, and Mer) of receptor tyrosine kinases that functions in homeostatic phagocytosis of apoptotic cells and negatively regulates TLRs and TLR-induced proinflammatory cytokine production (33–35). It remains unclear if human AS DCs represent a unique DC subset or a transitional cell type (36, 37). While *in vivo* roles are unknown, AS DCs are competent to activate CD4^+^ and CD8^+^ T cells and secrete IL-12p70 and IFNα when activated by TLR agonists *in vitro*. AS DCs are subdivided into AS DC1 (pDC-like; secrete IFNα) and AS DC2 (cDC-like; secrete IL-12p70) (15, 31).

Critical to DC development and homeostasis is the receptor tyrosine kinase Flt3 (15, 38). Murine models have established that cDC and pDC development, function, and maintenance is reliant on Flt3L (FL), but less is known concerning humans (39–42). In humanized mice, FL administration causes cDC expansion (43). Exogenous FL drives human HSPC *in vitro* differentiation into cDCs and administration of FL *in vivo* induces cDC expansion (44–47). Finally, individuals with a homozygous loss-of-function variant of *FLT3LG* have reduced BM DC progenitors and blood cDCs and pDCs (48). Thus, Flt3 signaling is also critical for human DC genesis and homeostasis.

A comprehensive study of blood DC subsets and BM DC progenitors in CLL is lacking and necessary to fully understand their immunodeficiency. cDC1, cDC2, and AS DCs have not been examined in CLL nor have BM DC progenitors. This study examined blood cDC1, CD5^−^ and CD5^+^ cDC2, AS DC1, AS DC2, and pDC in conjunction with paired serum FL and Flt3 expression in untreated CLL (UT-CLL) patients. In a separate cohort of UT-CLL with paired blood and BM samples, we investigated the status of BM DC progenitors to blood DC subsets to elucidate precursor-progeny relationships. Finally, we examined blood DCs in CLL patients undergoing BTK inhibitor (BTKi) acalabrutinib treatment and the relationship between DC deficiencies and clinical correlates.

## Materials and methods

### Patient samples

All patient or healthy control (HC) blood, BM and serum samples were cryopreserved after density gradient separation. Patient characteristics are detailed in **Sup. Tables 2** and **4**. Male and female patients were evaluated and HCs were age-matched. HC blood was obtained from apheresis cones. Unpaired HC serums were utilized as comparators for the FL and TNFα capture ELISA assays. The Mayo Clinic CLL Tissue Bank provided UT-CLL paired PBMC and serum samples. The patients selected for the study had signed informed consent to provide biobanked research samples (IRB protocol 1827–00). Cells were used immediately post-thaw and retained on ice. All patient and HC samples were divided into blinded randomized batches and representation of HC and CLL-IPI groups were included within each batch to prevent batch effects from confounding data analysis. Baseline (just prior to therapy) and 12-month response evaluation of acalabrutinib treated CLL samples were thawed together for comparison.

### Flow cytometry and analysis

Flow cytometry staining of PBMC and BM-MNC samples was done as described in Welch, et al. (49). FlowJo 10.5.3 (Becton Dickinson) was used for flow cytometry analysis. DC panel 1 was used on HC n=25/25, UT-CLL n=87/87, CLL BM paired PBMC n=15/26, and frontline acalabrutinib treated CLL n=12/12. DC panel 2 was used on HC n=16/25 and UT-CLL n=78/87. DC subsets in blood are reported as a percent of CD19^+^ CD5^+^ -excluded PBMC (12). Gates were batch-corrected and customized on an as-needed basis to accommodate individual human HC and CLL patient variation to accurately capture populations of interest. Isotype controls were used to confirm Flt3 surface expression and gating of AS DCs. For DC panel 3, isotype controls were used to define positivity for Flt3 and CD116 and FMO to define positivity for CD115.

### ELISA

Serum FL and TNFα quantification was completed according to kit instructions (R&D Systems Human FLT3L Quantikine ELISA Kit Cat. DFK00 and Human TNF-alpha Quantikine ELISA Kit Cat. DTA00D). Serum FL levels of 7/87 UT-CLL patients were below the ELISA kit minimum detectable dose (MDD) of 7 pg/mL and reported as this value (triangle symbol).

### Clinical correlates

Serum immunoglobulin (Ig) levels were obtained within 1.5 years of the UT-CLL paired PBMC/serum sample acquisition date. Reported infection data and serum Ig values were extracted from the Mayo Clinic patient care database by trained database personnel.

### Statistical analysis

Statistical tests are indicated in all figure legends and performed using GraphPad Prism version 9.1.0 (GraphPad Software, San Diego, CA, USA). Parametric unpaired t-tests were used to compare HC versus UT-CLL. Mann-Whitney tests were used comparing HC and UT-CLL BM DC progenitor frequencies and surface Flt3 expression. One-way ANOVA with multiple comparison Kruskal-Wallis tests were used to compare HC and multiple indicated UT-CLL groups. Paired Wilcoxon tests were used for comparing paired CLL patients at baseline versus 12-months of acalabrutinib treatment (HC are shown for reference). Time to events (i.e., first infection or first treatment) were calculated from the time of sample until the event, death, or last known alive date. Time-to-event analyses were visualized via cumulative incidence models adjusting for competing risk of death (Aalen-Johansen methodology) and analyzed via Cox proportional hazards (PH) regression models. Factors which were univariably significant were analyzed in a multivariable Cox PH model adjusting for CLL-IPI. Time to event analyses were conducted in SAS 9.4 (SAS Institute, Cary, NC).

## Results

### DC deficiencies in untreated CLL patient blood

PBMCs from 87 UT-CLL (median age 67 years; range (R): 35–88) and 25 age-matched healthy control (HC) (median age 64 years; R: 53–77) were evaluated. No statistical difference in frequencies of DCs between male and female patients was observed (50). Two unique flow cytometry panels were developed based on current advances in human DC subset characterizations: panel 1 characterized and quantified blood DC populations and panel 2 assessed functional proteins on cDCs **(Sup. Table 1)**. The UT-CLL cohort we studied was very representative of the span of CLL. Specifically, all Rai subgroups were represented, had a median age of 67, relatively equal percentages of mutated and unmutated *IGHV* subgroups, and all four CLL-IPI risk groups were represented **(Sup. Table 2)**. In addition, this cohort had a range of clonal CD19^+^ CD5^+^ B cells (CLL B cells) frequencies typically seen in UT-CLL **(Sup. Fig. 1A)**.

**Figure 1:**
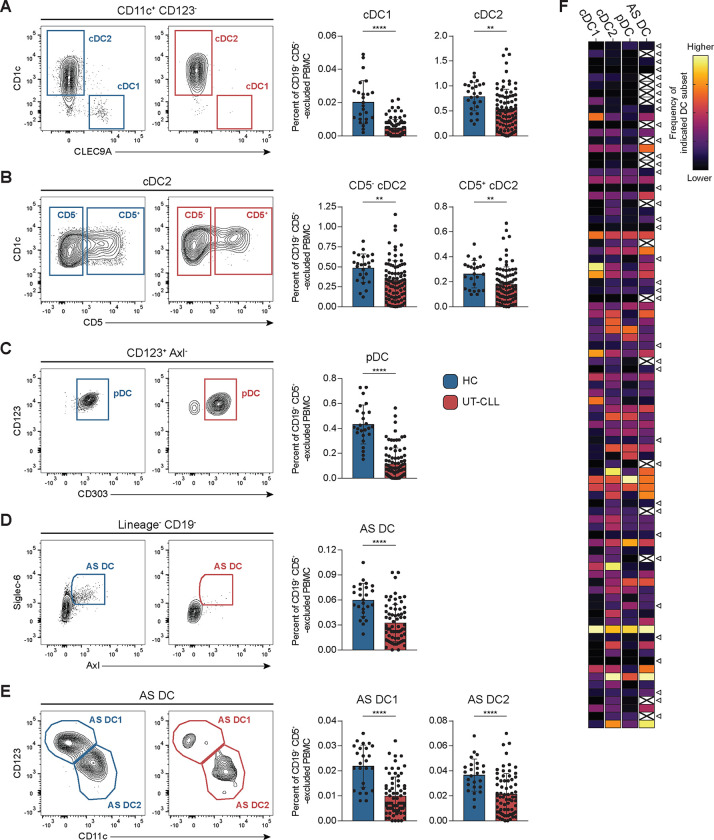
DC compartment deficiencies in untreated CLL patient blood. **(A-E)** Left: all representative flow cytometry plots are gated from CD19^+^ CD5^+^ -excluded Lin^−^ HLA-DR^+^ shown for the indicated DC subsets from UT-CLL and HC **(Sup. Fig. 1B, C)**. Right: the frequency of indicated DC subsets are reported as a percent of CD19^+^ CD5^+^ -excluded PBMC. **(A)** cDC1 (Lin^−^ HLA-DR^+^ CD123^−^ CD11c^+^ CLEC9A^+^) (65/87 UT-CLL had detectable cDC1) and cDC2 (Lin^−^ HLA-DR^+^ CD123^−^ CD11c^+^ CD1c^+^ CLEC9A^−^) **(B)** CD5^−^ and CD5^+^ cDC2 **(C)** pDC (Lin^−^ HLA-DR^+^ CD123^+^ CD11c^−^ CD303^+^) **(D)** AS DC (Lin^−^ HLA-DR^+^ Axl^+^ Siglec-6^+^) **(E)** AS DC1 and AS DC2. **(F)** Heatmap of relative frequency of the indicated DC subset (columns) as a percent of CD19^+^ CD5^+^ -excluded PBMC for the 87 UT-CLL patients (rows). Color is scaled on each of the following UT-CLL frequency ranges: cDC1 0–0.022%, cDC2 0–1.74%, pDC 0–0.564%, AS DC 0–0.093%. Triangle symbols indicate UT-CLL patients with shared low frequencies across all available DC subsets. Cells crossed out indicates data was excluded due to less than 85% of AS DC were contained within the AS DC1 and AS DC2 subpopulation gates. UT-CLL patients were sorted by increasing serum FL (top down). **(A-E)** Bar graphs indicate the mean with SD error bars. Statistical significance shown are as follows *p<0.05, **p<0.01, ***p<0.001, and ****p<0.0001.

**Sup. Fig. 1B-C** outlines our gating strategy used to distinguish phenotypic and functional DC populations: cDC1, cDC2 (CD5^−^ and CD5^+^ cDC2), pDC, AS DC1, and AS DC2. **Sup. Fig. 2** shows the unique combination of surface markers we employed to identify DC subsets and relative frequencies of DCs in HCs. To exclude monocyte-derived cells, CD14 and CD16 were included in the lineage cocktail of panels 1 and 2 (31, 51). To compare DC frequencies between UT-CLL and HC, a CD19^+^ CD5^+^ cell exclusion step was implemented or CD19 was included in the lineage cocktail, as we have previously described **(Sup. Fig. 1B-D**, **Sup. Fig. 3)** (12). Both cDC1 and, to a lesser but still significant extent, cDC2, are reduced in UT-CLL compared to HC (Fig. 1A). cDC2 consist of functionally distinct CD5^−^ and CD5^+^ cDC2 (27, 28). **Sup. Fig. 4** shows the abundance of CD5^−^ and CD5^+^ cDC2 in HCs. UT-CLL patients were found to have reduced CD5^−^ and CD5^+^ cDC2 (Fig. 1B).

pDCs are dysfunctional in CLL and previously found to be reduced in blood (8). Here, we investigated pDCs in a larger and more diverse UT-CLL cohort. Unique to this study, was removal of AS DCs before gating on pDCs, since AS DCs can skew pDC frequencies **(Sup. Fig. 1B-C)**. Fig. 1C confirms and extends the significant pDC reductions in UT-CLL blood. Notably, pDCs were the only DC subset that inversely correlated with frequencies of CLL B cells **(Sup. Fig. 5A)**.

Total AS DCs and AS DC1 and AS DC2 subsets were also evaluated. All three populations were significantly reduced in UT-CLL (Fig. 1D-E). In this analysis we note that 18 UT-CLL were excluded for the analysis due to insufficient cells within the AS DC1 and AS DC2 gates **(Sup. Fig. 1B-C)**.

These data reveal a broad DC deficiency in UT-CLL. Notably, 37/87 UT-CLL patients exhibited total DC subset deficiencies (Fig. 1F, **triangles and Sup. Fig. 5B-C)**.

### Altered expression of functional surface proteins in select DC subsets in UT-CLL

**Figure 2:**
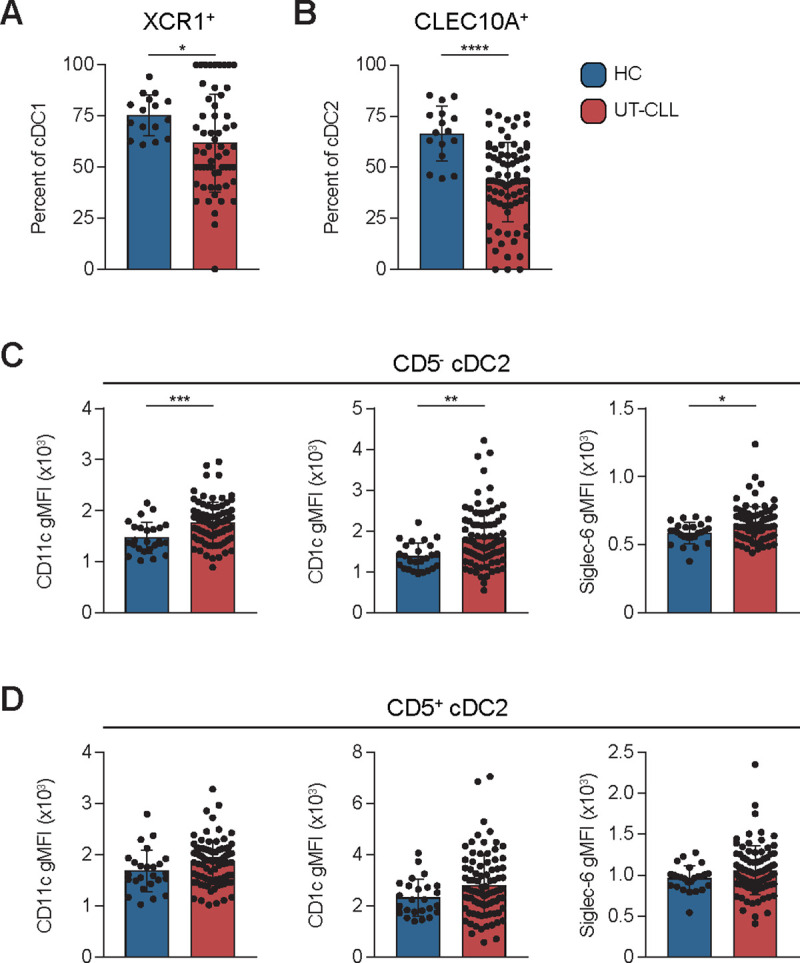
Altered expression of functional surface proteins in select DC subsets in UT-CLL. **(A)** The frequency of XCR1^+^ cDC1 for UT-CLL and HC reported as a percent of total cDC1 (58/78 UT-CLL had detectable cDC1). **(B)** The frequency of CLEC10A^+^ cDC2 for UT-CLL and HC reported as a percent of total cDC2. **(C)** The surface expression (gMFI) of CD1c, CD11c, and Siglec-6 on CD5^−^ cDC2 is shown for HC and UT-CLL. **(D)** The surface expression (gMFI) of CD1c, CD11c, and Siglec-6 on CD5^+^ cDC2 is shown for HC and UT-CLL. **(A-D)** Bar graphs indicate the mean with SD error bars. Unpaired t-tests are shown. Statistical significance shown are as follows * = p<0.05, ** = p<0.01, and *** = p<0.001, and **** = p<0.0001.

pDCs are known to be dysfunctional in CLL due to reduced expression of PAMP sensing molecules and secretion of IFNα (8). However, less is known about cDC dysfunction. Thus, expression of markers associated with important cDC functions were evaluated. XCR1 on cDC1 and CLEC10A and CD11c on cDC2 in UT-CLL were examined (Fig. 2). CD5^−^ and CD5^+^ cDC2 differentially express CD11c and were analyzed separately **(Sup. Fig. 4)**.

XCR1 is a chemokine receptor involved in chemotaxis towards XCL1 produced by activated CD8^+^ T cells (52–55). Frequencies of XCR1^+^ cDC1 in UT-CLL were reduced (p=0.0316) (Fig. 2A). CLEC10A is an endocytic CLR that when bound to ligand enhances TLR-7/8 induced cytokine secretion (29). Reductions in CLEC10A^+^ cDC2 were observed in UT-CLL (p<0.0001) (Fig. 2B). CD11c binds iC3b, mediates phagocytosis, and is involved in the capture of CD47-deficient “missing self” cells (56–58). We observed elevated expression of CD11c on CD5^−^ cDC2 and on a subset of CD5^+^ cDC2 (Fig. 2C-D, **left)**.

### Serum FL identifies cDC2 and pDC deficiencies and reveals an inverse correlation with Flt3 expression in UT-CLL.

**Figure 3:**
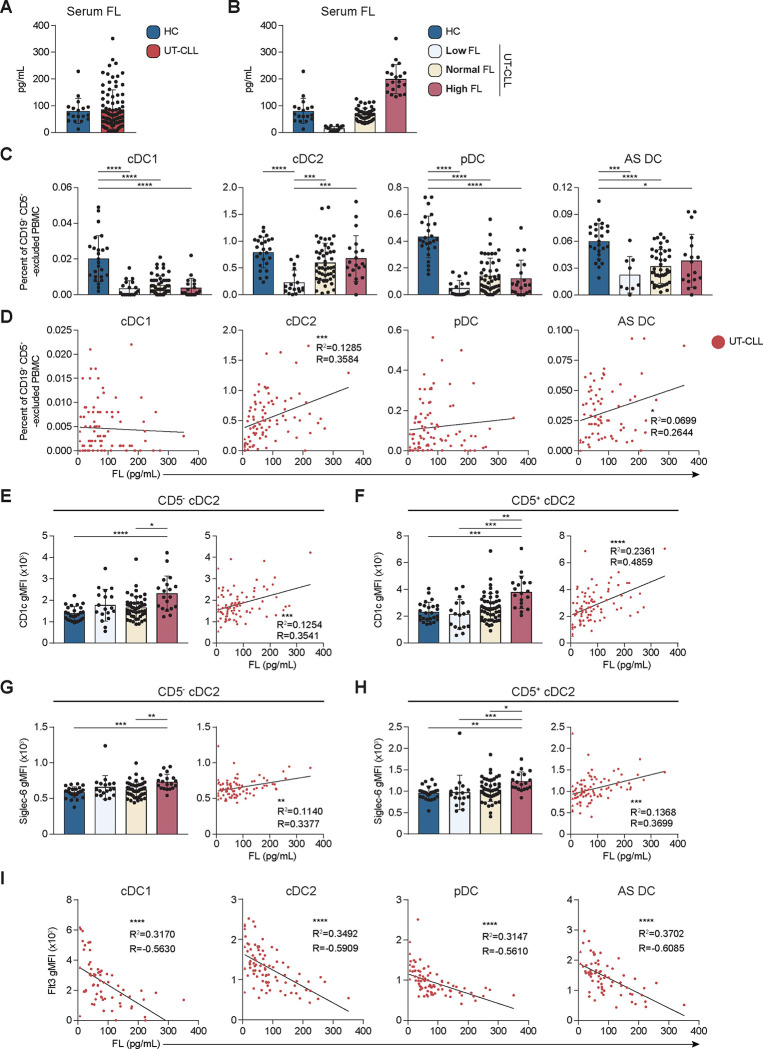
Serum FL identifies cDC2 and pDC deficiencies and reveals an inverse correlation with Flt3 expression in UT-CLL. **(A)** Serum FL concentrations (pg/mL) reported for UT-CLL (Mean (M)=91.38, Standard deviation (SD)=47.45) and HC (M=80.13, SD=70.53). **(B)** Serum FL groups in the UT-CLL cohort were determined as low (M=17.80, SD=7.58), normal (M=66.76, SD=24.46), and high FL (M=199.52, SD=54.41). HC are shown for reference. **(C)** The frequency of indicated DC subset shown as a percent of CD19^+^ CD5^+^ -excluded PBMC across serum FL group. **(D)** Correlation of indicated DC subset frequencies and the serum FL concentration in UT-CLL patients. **(E-H)** Left: indicated surface protein expression (gMFI) on CD5^−^ or CD5^+^ cDC2 between UT-CLL serum FL groups. Right: correlation of CD5^−^ or CD5^+^ cDC2 indicated surface protein expression and serum FL concentration (pg/mL) for UT-CLL. **(E and F)** CD1c. **(G and H)** Siglec-6. **(I)** Correlation of indicated DC subset surface Flt3 expression (gMFI) and the serum FL concentration (pg/mL) in UT-CLL patients. **(A-C, E-H, left)** Bar graphs indicate the mean with SD error bars. **(A-I)** Statistical significance shown are as follows * = p<0.05, ** = p<0.01, *** = p<0.001, and **** = p<0.0001.

The Flt3/FL signaling axis is critical to the development, homeostasis, and function of DCs (41, 42, 46, 48, 59, 60). Thus, we examined Flt3/FL and its relationship to DC subset frequencies in our diverse UT-CLL cohort. Serum samples isolated from the same whole blood collections as the PBMC samples analyzed for DC subsets were obtained. Serum FL concentrations were variable in our UT-CLL cohort compared to HC (Fig. 3A). Serum FL in our HC cohort were within the normal reported range (61). Using the HC serum FL distribution, we distinguished low or high FL UT-CLL patients as having a serum FL concentration greater than 1 SD below or above the mean of HC (Fig. 3B). Three serum FL groups were then established using this cutoff: low, normal, and high **(Sup. Table 3)**. Frequencies of CLL B cells were not significantly different between the serum FL groups **(Sup. Fig. 6A)**. Furthermore, serum FL concentration did not associate with frequencies of CLL B cells **(data not shown)**.

Each DC subset was evaluated according to UT-CLL serum FL group. cDC1 frequencies were reduced among all serum FL groups. cDC1 frequencies in the normal serum FL UT-CLL resembled HC (Fig. 3C). cDC2 were significantly reduced in the low serum FL group compared to HC (Fig. 3C and **Sup. Fig. 6B-C)**. pDC deficiencies we observed in all serum FL groups (Fig. 3C). While the reliance on Flt3/FL axis in AS DC remains to be elucidated, AS DC were also reduced across all serum FL groups (Fig. 3C and **Sup. Fig. 6D-E)**. Notably, only cDC2 frequencies positively correlated with serum FL concentration in our UT-CLL cohort (Fig. 3D and **Sup. Fig. 6F)**.

Functional cell surface proteins can be modulated by cytokine concentration. CD1c plays a role in presentation of lipid-based antigens to T cells and FL positively regulates CD1c expression (62, 63). We investigated if this dynamic was similar in the context of serum FL in cDC2s. CD5^−^ and CD5^+^ cDC2 CD1c expression was most elevated in high serum FL UT-CLL (Fig. 3E-F, **left**). Indeed, we identified a positive association between serum FL and CD1c expression on CD5^−^ and CD5^+^ cDC2 subpopulations (Fig. 3E-F, **right**).

Siglec-6 mediates extracellular vesicle uptake and recruits phosphatases to regulate cell signaling (64, 65). cDC2s express low levels of Siglec-6 **(Sup. Fig. 4B)**. Siglec-6 expression was elevated in high serum FL on CD5^−^ and CD5^+^ cDC2 (Fig. 3G-H, **left**). Like CD1c, Siglec-6 and serum FL concentration positively associated in UT-CLL on both CD5^−^ and CD5^+^ cDC2 subpopulations (Fig. 3G-H, **right**).

Flt3 is internalized upon ligation with FL (66–68). Thus, we evaluated the association between Flt3 expression on DCs and serum FL in UT-CLL. Flt3 expression on all DCs inversely correlated with increasing serum FL (Fig 3I and **Sup. Fig. 6B-E, G**). Thus, we document that serum FL is associated with Flt3 expression in UT-CLL.

TNFα is a negative regulator of Flt3 on pDC (8). Therefore, we evaluated if serum TNFα levels in UT-CLL influenced DC subset frequencies **(Sup. Fig. 7A and Sup. Table 3)**. TNFα levels had minimal to no association on DC subset frequencies or Flt3 expression in UT-CLL patients **(Sup. Fig. 7B-E)**.

### Deficiencies in bone marrow DC progenitors in UT-CLL patients.

**Figure 4:**
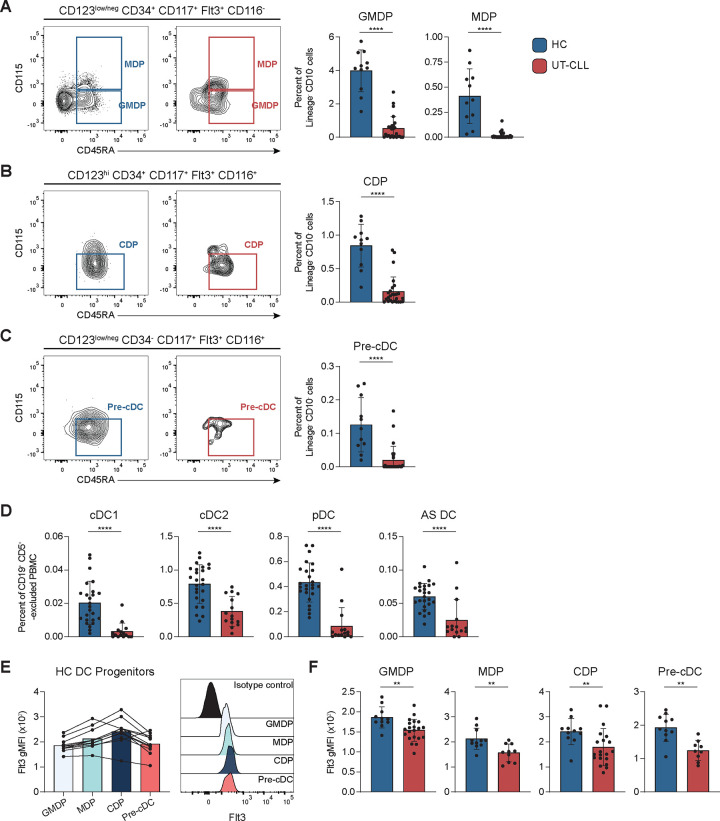
Deficiencies in bone marrow DC progenitors in UT-CLL patients. **(A-C)** All populations are CD45^+^ Lin^−^ CD10^−^ CD303^−^ CD141^low/−^. Left: representative flow cytometry plots of indicated BM DC progenitors from UT-CLL and HC shown in **Sup. Fig. 8** gating strategy. Right: frequency of indicated BM DC progenitors reported as a percent of Lin^−^ CD10^−^ mononuclear cells (MNC). **(A)** GMDP (CD123^low/−^ CD34^+^ CD117^+^ CD116^−^ Flt3^+^ CD115^−^ CD45RA^+^) and MDP (CD123^low/−^ CD34^+^ CD117^+^ CD116^−^ Flt3^+^ CD115^+^ CD45RA^+^). **(B)** CDP (CD123^high^ CD34^+^ CD117^+^ CD116^+^ Flt3^+^ CD115^−^ CD45RA^+^). **(C)** Pre-cDC (CD123^low/−^ CD34^−^ CD117^+^ CD116^+^ Flt3^+^ CD115^−^ CD45RA^+^). **(D)** Frequency of indicated DC subsets from 15/26 UT-CLL paired blood are reported as a percent of CD19^+^ CD5^+^ -excluded PBMC. **(E)** Left: quantification of the differential surface Flt3 expression (gMFI) on DC progenitors from HC individuals. Right: representative HC histogram showing Flt3 expression (normalized to mode) on GMDP, MDP, CDP, and Pre-cDC. **(F)** GMDP, MDP, CDP, and Pre-cDC Flt3 expression shown in UT-CLL and HC. **(A-F)** Bar graphs indicate the mean with SD error bars. Statistical significance shown are as follows ** = p<0.01 and **** = p<0.0001.

BM hematopoietic defects have been reported in UT-CLL, but BM DC progenitor alterations have not been reported (12). We hypothesized that impaired production of BM DC progenitors in UT-CLL may contribute to mature blood DC deficiencies. Panel 3 **(Sup. Table 1 and Sup. Fig. 8)** was adapted from Breton et al. to evaluate GMDP, MDP, CDP, and pre-cDCs in 26 UT-CLL and 11 age-matched HC BM samples (16). Clinical characteristics of the UT-CLL BM cohort are shown in **Sup. Table 4.** Frequencies of GMDP and MDP were reduced as a percent of CD45^+^ Lin^−^ CD10^−^ cells (GMDP; p<0.0001 and MDP; p<0.0001) (Fig. 4A). CDPs give rise to pre-cDCs and pDCs and were reduced in UT-CLL (p<0.0001) (Fig. 4B). Pre-cDCs were similarly reduced (p<0.0001) (Fig. 4C). 15/26 of the UT-CLL BM samples had paired PBMCs to allow investigation of precursor-progeny relationships. Panel 1 was used to determine if patients with BM DC precursor deficiencies had reduced frequencies of cDC1, cDC2, pDCs, and/or AS DCs in blood. Blood DC subsets were reduced in patients with BM DC progenitor deficiencies (Fig. 4D). We also examined Flt3 expression on DC progenitors and found reduced levels of Flt3 in all four BM DC progenitor subsets in UT-CLL (Fig. 4E-F). Taken together, these data strongly suggest that impaired BM hematopoiesis contributes to diminished DC subset frequencies in blood of CLL patients.

### High CLL-IPI identifies CLL patients with cDC deficiencies.

**Figure 5:**
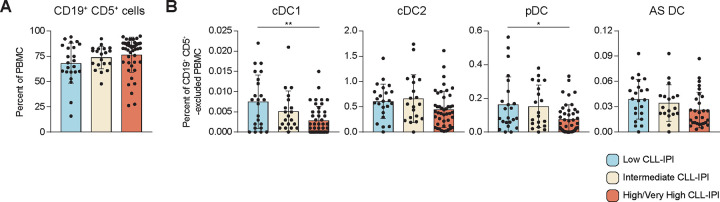
High CLL-IPI identifies CLL patients with cDC deficiencies **(A)** The frequency of CD19^+^ CD5^+^ CLL B cells are reported as a percent of PBMC across CLL-IPI group. **(B)** Indicated DC subsets are reported as a percent of CD19^+^ CD5^+^ -excluded PBMC across CLL-IPI group. **(A-B)** Bar graphs indicate the mean with SD error bars. Statistical significance shown are as follows * = p<0.05, ** = p<0.01, and **** = p<0.0001.

The CLL International Prognostic Index (CLL-IPI) is a highly accurate model to predict survival and time-to-first treatment (TTFT), integrating age, Rai stage, *IGHV* mutation status, serum β2-microglobulin levels, and chromosomal aberrations (69, 70). Patients are scored based on these features then categorized as: Low, Intermediate, High, or Very High risk. DCs were examined across CLL-IPI risk groups in our cohort. Frequencies of CLL B cells were similar between CLL-IPI groups (Fig. 5A). Only cDC1 and pDC were found to be reduced in High/Very High CLL-IPI compared to low CLL-IPI patients (Fig. 5B).

### Clinical manifestations of UT-CLL patients with shared DC subset deficiencies

**Figure 6:**
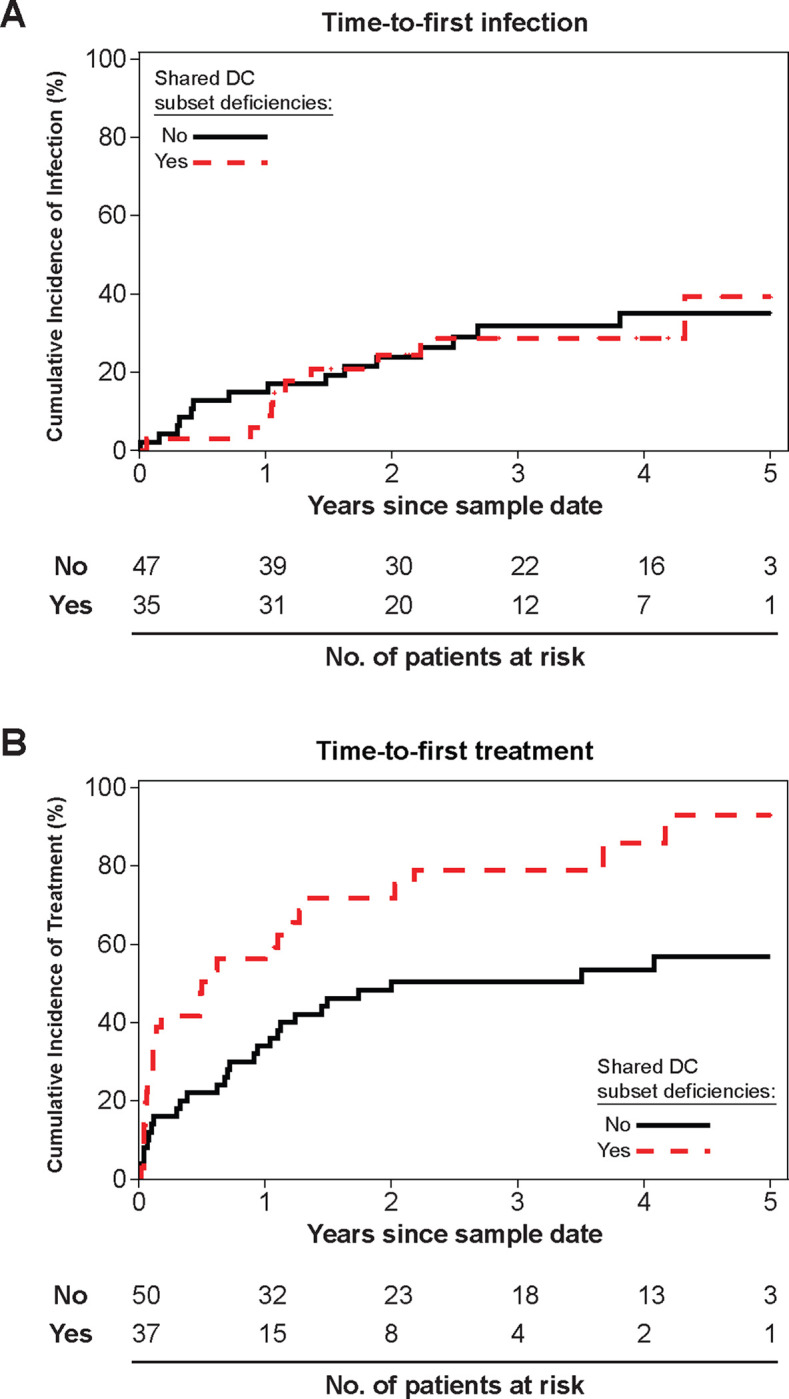
Clinical manifestations of UT-CLL patients with shared DC subset deficiencies. **(A and B) Sup. Table 6** contains statistical analysis results. **(A)** The cumulative incidence of infections according to shared DC subset deficiency group in the UT-CLL patients without known prior documented infections (n=82/87) as observed in **Fig. 1F (triangles) and Sup. Fig. 5B**. **(B)** The cumulative incidence of treatment according to shared DC subset deficiency group in the UT-CLL patients (n=87/87) as observed in **Fig. 1F (triangles) and Sup. Fig. 5B**.

CLL patients are susceptible to serious infections (4, 5). Since DCs provide frontline detection and protection against pathogens, we hypothesized that DC deficiencies associate with serious infections in UT-CLL **(Sup. Table 3 and 5)**. We focused on UT-CLL patients with (n=37/87) or without shared DC subset deficiencies (Fig. 1F, **triangles and Sup. Fig. 5B)**. We found no difference in time-to-first infection between DC deficiency groups in UT-CLL patients without a recorded infectious event prior to our PBMC/Serum sample date (n=82/87) (Fig. 6A and **Sup. Table 6)**.

Poor prognostics of unmutated IGHV status, elevated CD49d, CD38 and ZAP70 status associated with shorter TTFT were proportionally expanded in UT-CLL patients that eventually required treatment **(Sup. Fig. 9A)** (69, 71–73). We also determined that UT-CLL with shared DC subset deficiencies had shorter TTFT compared to UT-CLL without this reduction (p=0.0047) (Fig. 6B and **Sup. Table 6)**. The association held after adjusting for CLL-IPI (p=0.0158).

### pDC rebound in acalabrutinib treated CLL patients.

**Figure 7:**
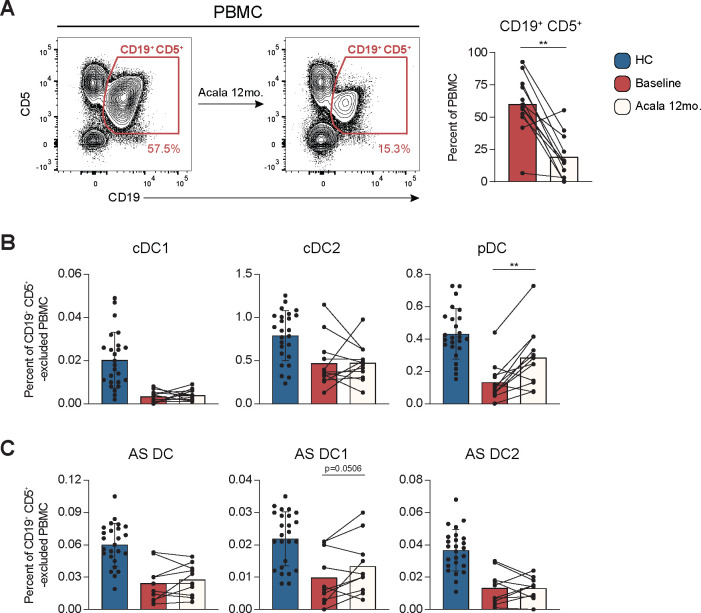
pDC rebound in acalabrutinib treated CLL patients. **(A-C)** Data from 12 CLL patients at baseline and 12-months of frontline acalabrutinib treatment. HC are shown for reference. **(A)** Left: representative flow cytometry plots of CD19^+^ CD5^+^ CLL B cells before and after acalabrutinib treatment at 12-months. Right: frequency of CD19^+^ CD5^+^ CLL B cells at CLL baseline and 12-months acalabrutinib treatment reported as a percent of PBMC. **(B and C)** The frequency of indicated DC subsets reported as a percent of CD19^+^ CD5^+^ -excluded PBMC. **(B)** Frequency of cDC1, cDC2, and pDC. **(C)** Frequency of AS DC, AS DC1, and AS DC2. **(A-C)** Bar graphs indicate the mean with SD error bars. Statistical significance shown are as follows ** = p<0.01.

We recently reported that myeloid DC and pDCs were normalized in CLL patients treated with the BTKi ibrutinib (49). Here, we investigated if DC subsets had a similar response in patients treated with the BTKi acalabrutinib (74–77). In a cohort of 12 patients, 10/12 were responsive to acalabrutinib after 12 months of therapy (Acala 12mo.) (Fig. 7A and **Sup. Table 4)**, manifested as a 64.45% average reduction in blood CLL B cells frequencies. Next, we examined cDC1, cDC2, pDC, and AS DC at Acala 12mo. Only pDC frequencies increased at Acala 12mo, confirming our original study of ibrutinib treated CLL. Importantly, 8/12 patients had pDCs in the HC range (Fig. 7B, **right**). AS DC1 frequencies were also increased at Acala 12mo. approaching significance (p=0.0506) (Fig. 7C, **middle**). No consistent change was observed in cDC1 or cDC2 at the Acala 12mo. timepoint (Fig. 7B-C).

## Discussion

Canonical DCs, comprised of cDC1, CD5^−^ and CD5^+^ cDC2, pDC, and AS DC, with specialized functions and roles in immunity, have yet to be fully examined within a cohort of CLL patients with diverse disease characteristics. Here, we report broad blood and BM DC progenitor deficiencies in UT-CLL patients who had clinical and prognostic features typically seen in this disease. Flt3/FL signaling is critical in DC biology. We establish that serum FL identifies cDC2 and pDC deficiencies that correlate with Flt3 expression. Importantly, patients with a shared DC subset deficiency had a significantly shorter TTFT. Acalabrutinib therapy was found to selectively restore pDCs in CLL. Collectively, this comprehensive study provides new insight into the nature of immunodeficiency in UT-CLL and can be utilized as a modern framework to uncover associations with CLL disease status and its complications.

cDC1 are critical players in anti-tumor and intracellular pathogen immune responses. They are highly proficient cross-presenters of exogenous antigen on MHC class I (53, 54, 78). Activated CD4^+^ T cell licensing of cDC1 results in optimally activated CD8^+^ T cells (52, 79). We identified significant cDC1 deficiencies in UT-CLL. High/Very High CLL-IPI risk patients had further reduced cDC1 frequencies than Low CLL-IPI risk patients. This adds to the clinical utility of the CLL-IPI prognostic model since it informs us of this component of the immune system. Indeed, our work on the CLL-IPI model has found an increased risk of infections in High and Very High CLL-IPI risk cohorts (Sameer Parikh, unpublished observation). While we did not observe clear associations of serious infections with the DC frequencies we believe that additional work with a larger number of CLL patients will be needed.

cDC2 were reduced in UT-CLL, but most variable among the evaluated DC subsets. CD5 expression on cDC2 is associated with robust CD4^+^ and CD8^+^ T cell priming. CD5^+^ cDC2 can prime CD5^hi^ T cells in the context of immune checkpoint blockade therapy, facilitating tumor rejection (28, 80). We found that CD5^−^ and CD5^+^ cDC were reduced. Notably, checkpoint blockade therapy has shown generally poor efficacy in CLL compared to other cancers (81). Additional studies are needed to determine if reductions in cDC2 may be able to stratify CLL patient responsiveness to checkpoint blockade therapy. In particular, this aspect is a potential mechanistic explanation of how effective a given CLL patient’s T cells are in the context of their anti-leukemic function.

pDCs are also reduced in UT-CLL and we confirmed that finding in this study (8). In this study, we examined pDCs across the span of CLL disease demographics given the typical clinical and prognostic heterogeneity of our CLL cohort as well as by using CLL-IPI risk groups (31). High/Very High CLL-IPI risk patients were found to harbor more severe pDC deficiencies than low CLL-IPI patients. Notably, only pDCs were normalized upon CLL B cell debulking with acalabrutinib therapy adding to the evidence that BTKi may enhance the immune system in important ways (82, 83). Our work suggests that pDCs may be subject to crowding out in various sites including bone marrow by extensive tissue site infiltration of CLL B cells.

One study reported elevated serum FL levels in UT-CLL (8). In this study, we documented variable levels of serum FL in our diverse UT-CLL cohort relative to HCs. To more fully dissect out how serum FL levels related to blood DC profiles, patients were segregated by low, normal, and high serum FL, then frequencies of DC subsets and their expression of Flt3 evaluated. Low serum FL significantly influenced the abundance of cDC2 in UT-CLL. In contrast, FL was not predictive of cDC1 frequencies despite high levels of Flt3 expression, which may result because cDC1 are not as reliant on FL during the final stages of development (84). Interestingly, Flt3 expression on all DC subsets, including AS DC, inversely correlated with serum FL in CLL. Activated CD4^+^ T cells are the primary source of FL in the periphery (40). T cell exhaustion is well established in CLL, but a link between their exhaustion status, serum levels of FL, and cDC2 prevalence has not yet been investigated. Additional studies are warranted to investigate these immunologic associations. Given the association of serum FL with cDC2 frequencies, future studies that provide maneuvers to enhance circulating FL levels in CLL may be of clinical interest.

Reductions in XCR1^+^ cDC1 and CLEC10A^+^ cDC2 may have functional implications for susceptibility to viral infections and tumor surveillance in CLL. CD11c and Siglec-6 contribute to cDC2 phagocytosis and extracellular vesicle uptake while CD1c is involved in presentation of lipid-based antigens to T cells (58, 62, 64). We hypothesized that serum FL levels might account for the variable expression of these markers. Indeed, the high serum FL cohort identified elevated CD1c and Siglec-6 on cDC2s. Thus, serum FL may be a novel biomarker for cDC2 functionality in UT-CLL.

cDCs are short-lived and require continuous replenishment from BM hematopoiesis (15, 17, 85). Because of this we studied and identified reduced frequencies of BM progenitors that generate cDCs and pDCs, suggesting a direct precursor-progeny relationship (12). Furthermore, Flt3 expression was reduced on GMDP, MDP, CDP, and pre-cDC in UT-CLL compared to HC, suggesting alterations in Flt3 signaling may contribute to reductions in DC progenitors. This finding is unique and provides an explanation for the blood DC deficiencies in CLL. TNFα is a negative regulator of Flt3 (8). We previously showed that CLL-derived TNFα impairs BM myelopoiesis (13). Thus, it is interesting to speculate that local production of TNFα by CLL B cells may impair DCpoiesis in BM by altering expression of Flt3.

In summary, this first in kind, comprehensive study, uncovered broad DC subset deficiencies in UT-CLL patients. These findings were ascertained in a cohort that reflects the typical heterogeneity of CLL patients. In addition, these studies found that some DC deficiencies encompass CLL-IPI defined correlates of disease prognosis and progression and response to therapy. We also uncovered that serum FL levels may be a useful biomarker for DC deficiency and that administration of FL could be of clinical benefit for CLL patients both to correct the abnormal DC hematopoiesis and to improve T cell function. The reversibility of deficient pDCs by BTKi suggest current therapies may be of clinical benefit for DC correction but need to be further studied. Of high clinical significance, UT-CLL patients with deficiencies across all DC subsets (shared DC deficiency) had shorter TTFT. This study provides a novel and comprehensive platform for future studies of DCs in untreated and treated CLL patients and other hematologic malignancies with associated immunodeficiency.

## Abbreviations used in text and figures:

AS DC: Axl^+^ Siglec-6^+^ DC
BM: Bone marrow
cDC: conventional DC
CDP: common DC progenitor
CLL-IPI: CLL International Prognostic Index
DC: Dendritic cell
FL: Flt3 ligand
GMDP: granulocyte, monocyte, and DC progenitor
gMFI: geometric mean fluorescence intensity
HSPC: hematopoietic stem and progenitor cell
MC: Monocyte-derived cell
MDP: monocyte and DC progenitor
*IGHV*: Immunoglobulin heavy chain variable
pDC: plasmacytoid DC
Pre-cDC: pre-conventional DC
UT-CLL: Untreated Chronic Lymphocytic Leukemia
